# Editorial for the Bioinformatics of Human Diseases Special Issue

**DOI:** 10.3390/genes16020118

**Published:** 2025-01-22

**Authors:** Hongyan Xu

**Affiliations:** Department of Biostatistics, Data Science and Epidemiology, School of Public Health, Augusta University, Augusta, GA 30912, USA; hxu@augusta.edu

Bioinformatics plays an ever-increasing role in revealing the complexity of genomic information and how it is related to the susceptibility and pathophysiology of human diseases. With the availability of genomic data at primary DNS sequence, epigenomics, proteomics, and metabolomics levels, bioinformatics is essential in the analysis of the genomic data to illuminate the structure, function, interaction, and relationship between genes, proteins, and metabolites and how they play together in various human diseases. These discoveries will eventually lead to improved prevention and treatment of human diseases. This Special Issue, entitled “Bioinformatics of Human Diseases”, focuses on the recent development of bioinformatics methods for human disease identification, classification, diagnosis, and prognosis. This Special Issue contains six original research articles on novel bioinformatics analyses of data from a wide range of human diseases.

Steinkellner et al. [1] presented their work of using differential gene expression analysis, pathway enrichment analysis, and weighted gene co-expression network analysis (WGCNA) to identify nucleolin-related regulatory pathways in a human hepatoblastoma (HepG2) cell line. A total of 44 differentially expressed genes were identified between the siRNA cell models. The pathway enrichment analysis confirmed the essential role of nucleolin in DNA replication and cell cycle processes with the discovery of seven key genes implicated in DNA replication, cell cycle progression, and oncogenesis. These findings help improve our understanding of the molecular and pathologic mechanisms of nucleolin and new therapeutic perspectives in hepatoblastoma.

Haykal et al. [2] used several bioinformatics methods, including visualization of phylogenetic trees, 3D rendering, and the assessment of mutational impact, to analyze the mutation trend in various receptor-binding domains (RBDs) of the severe acute respiratory syndrome coronavirus 2 (SARS-CoV-2) spike protein from Indonesia published in the Global Initiative on Sharing All Influenza Data (GISAID) database. They identified 25 unique SARS-CoV-2 clades and 318 unique SARS-CoV-2 RBD mutations from the earliest COVID-19 sample to samples collected in 2022. Through the genetic profiling of the RBD sequences of various SARS-CoV-2 variants, they revealed a decreasing trend in virus pathogenicity as a potential trade-off to increase transmissibility via mutations in RBD over time.

Rapier-Sharman et al. [3] presented their work on the cause of high co-occurrence between systemic lupus erythematosus (lupus) and B-cell lymphoma (lymphoma). This is based on a novel immune imbalance transcriptomics (IITs) algorithm and RNA-sequencing (RNA-seq) data from lupus, lymphoma, and healthy B cells. They identified 344 IIT genes as known targets for current drugs. They also identified 296 novel lupus targets and 193 novel lymphoma targets. The known disease drug targets validate that IIT is an effective approach to identify disease genes. The findings contribute to the development of immune-related therapeutics for lupus and lymphoma.

Gregoris et al. [4] presented their work on the bioinformatics analysis of the aryl hydrocarbon receptor (AHR) and hypoxia-inducible factors (HIFs) pathway in developing clear cell renal cell carcinoma (ccRCC). They used protein–protein interaction network analysis and gene expression profiling in the analysis. They identified several proteins interacting with AHR significantly associated with poor survival rates in ccRCC, which could serve as novel therapeutic targets.

Xu et al. [5] presented their work on the identification of biomarkers and prediction models on Alzheimer’s disease (AD). They performed differential gene expression analysis on the datasets from the GEO database and cross-referenced them with Genecards to identify differentially expressed autophagy-related genes (DEAGs). GO and KEGG enrichment analyses and protein–protein interaction analyses were then performed, followed by experimental validation in a cell line. Three hub genes (TFEB, TOMM20, and GABARAPL1) were identified as potential biomarkers. Based on these biomarkers, they constructed a prediction model with good predictability. Their findings shed light on the mechanism and diagnosis of AD.

Archana et al. [6] investigated the functional consequences of non-synonymous SNPs (nsSNPs) in the ANTXR2 gene on antigen binding and their relationship with anthrax resistance. They performed computational analyses using Mutpred2 on the predicted function of the SNPs. Several deleterious mutations were identified, which are implicated in blood pressure regulations. These SNPs are worthy of further investigations to establish their implications for anthrax and other autoimmune disorders in humans.

The articles included in this Special Issue cover the bioinformatic analysis of a wide range of human diseases and provide comprehensive insights to direct future research of human disease using bioinformatics approaches. The bioinformatic methods are novel, and the findings from these analyses provide important leads for further studies to decipher the pathophysiology of the diseases. We anticipate that this Special Issue will help researchers refine our understanding of the disease etiology.

## Data Availability

Not applicable.
